# Correlates of pre-exposure prophylaxis amongst transgender women of color taking gender-affirming hormone therapy: Findings from the TURNNT cohort study

**DOI:** 10.1080/26895269.2026.2663935

**Published:** 2026-05-14

**Authors:** Eva Laura Siegel, Asher Baron, Alexander Furuya, Dustin T. Duncan, Lauren C. Houghton

**Affiliations:** Columbia University, New York, NY, USA

## Abstract

**Background:**

The Trying to Understand Relationships, Networks, and Neighborhoods Among Transgender Women of Color (TURNNT) Cohort Study provides a unique opportunity to identify correlates of PrEP use amongst transgender women of color taking gender-affirming hormone therapy (GAHT).

**Methods:**

We analyzed baseline data from the TURNTT Cohort Study, restricting our sample to those taking GAHT. We examined individual, interpersonal, and structural characteristics in association with current PrEP use using bivariate and multivariable models.

**Results:**

Healthcare factors played a critical role amongst those reporting GAHT use, with lower provider knowledge of transgender care and poor access to gender-affirming services associated with decreased likelihood of PrEP use; e.g., participants with only “okay” or “poor” access had 45% lower likelihood of PrEP use than those with “great” or “good” access (aPR = 0.55; 95% CI: 0.32–0.95). Gender and sexuality-related factors also predicted inequities, as individuals identifying with an additional gender (e.g., femme non-binary) had 70% lower likelihood of PrEP use than those identifying solely as transgender women (aPR = 0.30; 95% CI: 0.13–0.72), and those reporting higher sexual risk behaviors (such as having 6+ partners in the past six months) had more than double the likelihood of PrEP use compared to those with 0–1 partner (aPR = 2.28; 95% CI: 1.10–4.75).

**Conclusions:**

The findings suggest that both individual sociodemographic characteristics as well as interpersonal and structural healthcare-related characteristics play a significant role in shaping PrEP use among transgender women of color taking GAHT. This highlights the need for multilevel interventions that address structural and interpersonal determinants of health access to promote equity in HIV prevention.

## Introduction

HIV disproportionately burdens transgender women of color (TWOC) in the United States. One in five transgender women overall are living with HIV, over 34 times the rate in the general US population (Baral et al., 2013; Stutterheim et al., 2021). Among Black and Latina transgender women, HIV prevalence is estimated to be even higher, at around 44% and 28%, respectively (Becasen et al., 2019). These inequities are rooted in the intersectional oppressions that impair the lives of TWOC, including racism, transphobia, and misogyny (Crenshaw, 1991; LaMartine et al., 2024). Consequently, a range of multi-level determinants increase vulnerability to HIV in this population, and we explore these using a social ecological model (Baral et al., 2013; Duncan et al., 2019). Structural factors impacting HIV prevalence in TWOC include high rates of poverty, housing instability, food insecurity, sex work, and incarceration (Chen et al., 2020; Erickson et al., 2019; Poteat et al., 2019; Sevelius et al., 2021; Wilson et al., 2018). Interpersonal factors also contribute to HIV vulnerability: experiences of healthcare provider insensitivity or ignorance of transgender health, alongside language barriers in the healthcare setting, have been associated with lower engagement with HIV preventive care among TWOC (Nieto et al., 2021; Sevelius et al., 2016; 2021).

While HIV pre-exposure prophylaxis (PrEP) can be an effective HIV prevention method, uptake and consistent use are suboptimal among TWOC (Morris et al., 2024). Because of the elevated prevalence and incidence rate of HIV among TWOC, the CDC recommends that all TWOC without HIV be on PrEP to prevent HIV infection (Morris et al., 2024). Current estimates of use among TWOC range from 15–32% (Hood et al., 2018; Morris et al., 2024; Wilson et al., 2020). Given this, it is crucial to understand the factors influencing PrEP uptake (or lack thereof) in this population. Previous research has found that TWOC experience increased discrimination in attempting to access, and while accessing, healthcare compared to white transgender women. This results in their avoidance of the medical system due to anticipated discrimination (Bradford et al., 2013; Kattari et al., 2015; Lacombe-Duncan, 2016). Qualitative research demonstrates that cultural mistrust of medicine is a barrier to PrEP use among Black and Latina transgender women (Nieto et al., 2021; Poteat et al., 2019). In addition, some transgender women voice concerns about potential interactions between gender-affirming hormone therapy (GAHT) and PrEP. These concerns may lower their willingness to use PrEP (Hood et al., 2018; Jaiswal et al., 2021; Neumann et al., 2017; Poteat et al., 2019; Sevelius et al., 2016; Wilson et al., 2020) and have resulted in some transgender women discontinuing PrEP (Furuya et al., 2025).

Use of GAHT by transgender women is high: national estimates reported a prevalence of 72% in 2019–2020 (Eustaquio et al., 2024). Some researchers have found that access to gender-affirming care facilitates PrEP uptake (Cooney et al., 2022; Morris et al., 2024; Sevelius et al., 2016), and most transgender women are currently taking GAHT prescribed by a healthcare provider (Olansky et al., 2024). Positive experiences with gender-affirming, trans-competent providers make individuals feel more empowered about their own healthcare and more comfortable discussing and initiating PrEP (Sevelius et al., 2021; Storholm et al., 2024). However, we lack evidence on characteristics associated with PrEP use amongst TWOC taking GAHT that could inform interventions to increase PrEP uptake.

The Trying to Understand Relationships, Networks, and Neighborhoods Among Transgender Women of Color (TURNNT) Cohort Study provides a unique opportunity to identify correlates of PrEP use within a cohort of transgender women of color all taking GAHT. In an effort to deepen our understanding of the individual, interpersonal, and structural factors that contribute to PrEP uptake, we examine sociodemographic characteristics, gender/sexuality characteristics, behavioral characteristics, and healthcare-related characteristics in association with PrEP use amongst TWOC taking GAHT.

## Methods

### Sample

We analyzed baseline data from the TURNNT study, a 1-year longitudinal cohort study (2020–2022) aiming to understand the social and environmental structures impacting the health of transgender women of color in New York City. Designed in close collaboration with a Community Advisory Board, study staff administered 6 virtual quantitative surveys over Zoom in English and Spanish: two at baseline (Wave 1), two at six months (Wave 2), and two at one year (Wave 3) (Callander, 2020; Duncan et al., 2024). Surveys collected information on general health and well-being, socioeconomic characteristics, gender affirmation, sexual health and behaviors, sex work, and substance use. Study surveys were hosted using the online survey platform Qualtrics. Participants were compensated with pre-paid gift cards as follows: $50 per interview at enrollment (Wave 1), $75 at the 6-month stage (Wave 2), and $100 per interview at the 12-month stage (Wave 3). Survey administration at baseline took an average of 75 min.

Participants were mailed an HIV test between the first and second interviews at each wave and completed the test over a video call with the interviewer. All participants with preliminary positive test results were immediately directed to confirmatory blood testing with their primary care provider or with TURNNT partner organizations (Callander, 2020). Participants who reported an unknown or negative HIV status completed a point-of-care OraQuick saliva HIV test at the second interview of each wave in accordance with guidelines provided by the New York State Department of Health.

Of the TURNNT study’s full sample (*n* = 314), 88% (*n* = 277) reported taking GAHT. Those living with HIV at baseline were excluded from our analysis. We included 136 participants who were HIV negative/unknown at baseline and reported GAHT use. Forty-three percent (*n* = 59) of participants who reported GAHT use were currently taking PrEP.

### Eligibility and recruitment

The TURNNT study inclusion criteria were: (1) identifying as a transgender woman of color (TWOC), (2) being between 18 and 55 years old at enrollment, (3) living in the New York City metropolitan area, (4) speaking English or Spanish, and (5) planning to stay in the NYC metro area for at least 1 year. Study staff recruited participants through social media marketing, print advertising in community spaces, referrals from organizational outreach, and snowball sampling from enrolled participants. In total, 314 TWOC enrolled and completed baseline visits between August 2020 and November 2022 (more details are in Duncan et al., 2024). The Institutional Review Board at Columbia University Irving Medical Center (PT-AABQ1129) approved the study.

### Measures

#### Outcome:

We determined GAHT use by asking the question “I am going to read a list of gender affirming processes. For each one, tell me whether you have had or done it, want to have it done someday, or you do not want it: Hormone therapy”. There were three response options: (1) Have had or done it, (2) Want to have or do some day, (3) Do not want. We collapsed “Want to have or do some day” and “Do not want” into “Have not had” due to small cell counts (further details in Merriman et al., 2025). Of the participants who were HIV negative or unknown and therefore potentially taking PrEP, only *n* = 9 reported not taking GAHT; this subgroup was excluded from our analysis.

Participants who reported their HIV status as Negative or Unknown were asked about their PrEP use. To confirm that participants knew what PrEP was, they first answered the question: “PrEP (pre-exposure prophylaxis) is a once-daily pill that, when taken regularly by HIV negative people, can dramatically reduce the likelihood of infection. It is intended for regularly, long-term use. Before today, had you ever heard of PrEP?” Then, participants answered: “Have you ever taken PrEP to reduce the risk of getting HIV? If so, are you taking PrEP now?” The response options were: (1) No, never, (2) Yes, taking PrEP now, and (3) Yes, have taken PrEP previously but am not currently. Because our analysis aimed to understand participants’ PrEP use at the time of data collection, responses were recoded as “Yes, currently taking” and “No, not currently taking”.

#### Predictors:

We systematically selected all survey questions that were conceptually aligned with potential correlates of current PrEP use amongst those reporting GAHT use. We included individual, interpersonal, and structural factors; selected predictors fell within 4 categories: sociodemographic (education level, current housing status, race/ethnicity, nativity, language spoken at home, religiosity, age, sex work history, food insecurity, incarceration history), health and healthcare-related (self-rated health, insurance status, unmet medical need over past 6 months, having a primary care provider, primary care provider’s knowledge of transgender issues, having a doctor for transgender-related care, access to transgender care, having ever been denied transgender care by a doctor, having ever been denied transgender care by insurance), gender/sexuality (gender identity, sexual orientation, name legally changed, sex marker legally changed, living as transgender full-time, age at transition, and completion of gender-affirming medical interventions [breast augmentation, silicone injection, bottom surgery, orchiectomy, facial feminization surgery]), and behavioral (alcohol use/frequency, drug use/frequency, history of sexual assault, history of intimate partner violence, having ever had an STI, frequency of sex work, condom use during sex work, condom use (not in sex work), and number of sexual partners in the past 6 months). We also derived a ‘sexual risk’ predictor based on condom use and the number of sexual partners in the past 6 month. We derived this predictor following previous research that has adapted CDC PrEP prescribing guidelines to assess HIV acquisition risk among transgender women (Cooney et al., 2022; Malone et al., 2021).

### Statistical analysis

We calculated descriptive statistics for all predictors at baseline. We used modified Poisson regression analysis to obtain prevalence ratios (PR) (Zou, 2004) for current PrEP use among those reporting GAHT use, with those not reporting current PrEP use as the reference group. PR above 1.0 indicate increased likelihood of current PrEP use amongst those reporting GAHT use. Based on the literature and this dataset (Furuya et al., 2025), we ran both unadjusted models as well as adjusted models that included age, sexual orientation, income, and stable housing as covariates. In addition, we conducted a sensitivity analysis where we defined PrEP use as ever versus never.

## Results

**Figure 1.**
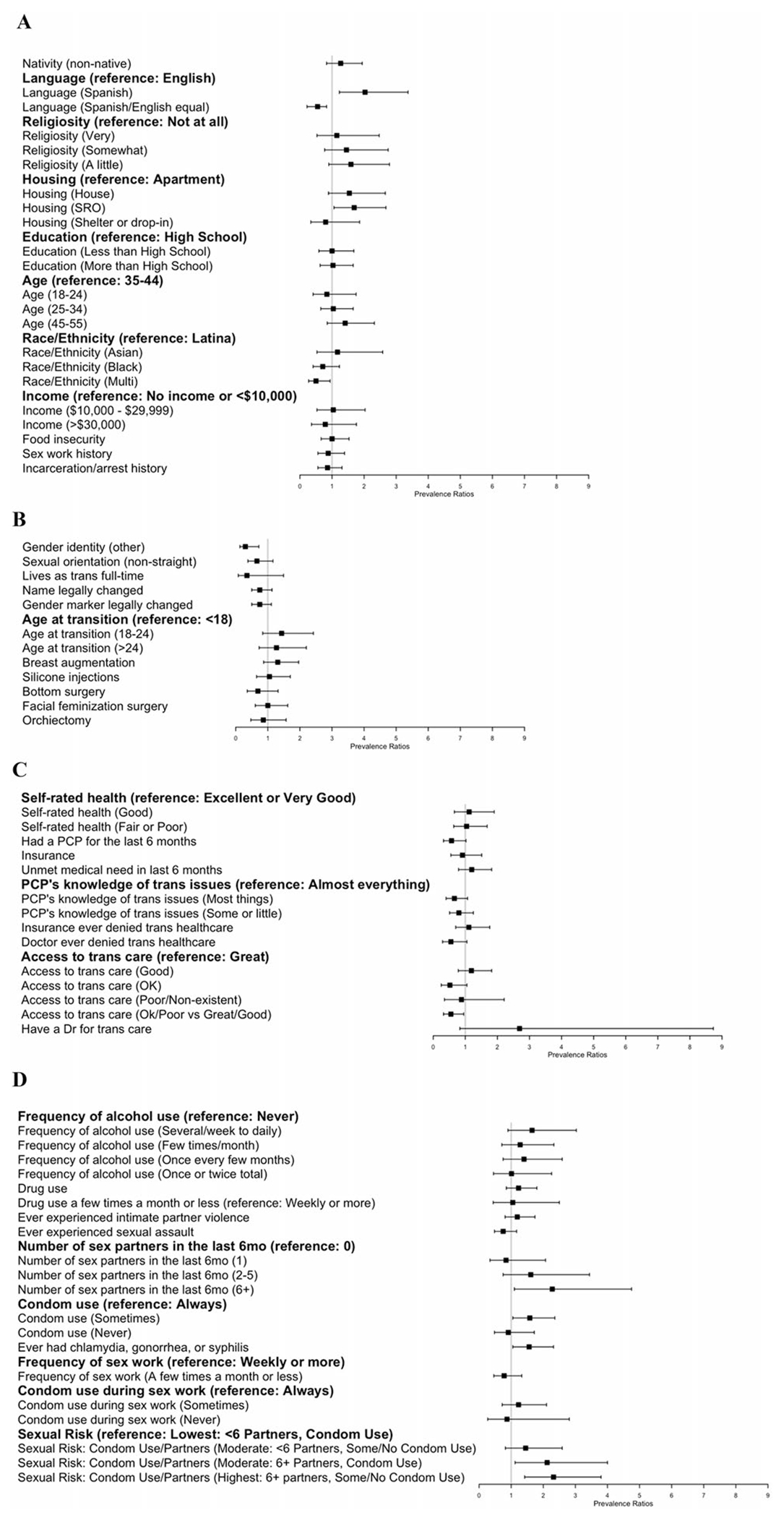
Adjusted prevalence ratios (PR) predicting current PrEP use amongst those reporting GAHT use, the TURNNT cohort study: demographic (panel A), gender/sexuality (panel B), health and healthcare (panel C), and behavior predictors (panel D). See supplementary tables for details.

Table 1 shows descriptive statistics for our analytic sample (*n* = 136). Figure 1 shows unadjusted and adjusted results from our modified Poisson regression models assessing associations between sociodemographic, health, gender/sexuality, and behavioral characteristics and current PrEP use among those who reported taking GAHT.

### Sociodemographic predictors

Participants who spoke Spanish at home had a higher likelihood of current PrEP use compared to participants who spoke English at home (adjusted Prevalence Ratio (aPR)=2.03; 95% CI: 1.23–3.37). Compared to those who identified as Latina, those who identified as multiracial were less likely to currently use PrEP (aPR = 0.50; 95% CI: 0.27–0.94). Participants living in a single room occupancy had 1.69 times the likelihood of current PrEP use compared to participants living in an apartment (aPR = 1.69; 95% CI: 1.06–2.68). We found that the likelihood of current PrEP use trended positively with age at time of data collection, for those who reported transitioning after age 18 (vs. <18), and for those who described themselves as any level of religious (vs. not religious at all) (Figure 1).

### Healthcare-related predictors

Access to gender-affirming care predicted current PrEP use. Compared to participants reporting great or good access to gender-affirming care, people with okay or poor access were less likely to be currently taking PrEP (aPR = 0.55; 95% CI: 0.32–0.95). Similarly, participants whose doctor had ever denied transgender care trended less likely to be currently taking PrEP (aPR = 0.55; 95% CI: 0.29–1.05).

### Gender/sexuality predictors

Compared to those who identified solely as transgender women, participants who identified as an additional gender (e.g., femme non-binary) were significantly less likely to report current PrEP use (aPR 0.3; 95% CI: 0.13–0.72). Similarly, participants who identified as any sexuality other than heterosexual trended less likely to report current PrEP use (aPR = 0.66; 95% CI: 0.38–1.16). Those who had completed a legal transition (legal name and gender change) also trended less likely to report current PrEP use compared with those who had not (legal name change: aPR = 0.75; 95% CI: 0.5–1.13; legal gender change: aPR = 0.75; 95% CI: 0.5–1.13).

Completion of gender-affirming medical interventions was marginally associated with current PrEP use but differed depending on the procedure. Participants who had completed breast augmentation or silicone injections trended more likely to report current PrEP use compared with those who had not (aPR = 1.31; 95% CI: 0.87–1.96). The reverse trend was observed for those who had completed bottom surgery (either ‘bottom surgery’ generally or the specific procedure orchiectomy) (bottom surgery: aPR = 0.69; 95% CI: 0.36–1.32; orchiectomy: aPR = 0.86; 95% CI: 0.47–1.57).

### Sexual behavioral predictors

Some sexual behaviors predicted current PrEP use. In general, the likelihood of current PrEP use increased with increasing sexual risk, with the highest sexual risk group 2.32 times more likely to report current PrEP use than the lowest sexual risk group (aPR 2.32; 95% CI: 1.42–3.8). Participants who reported having 2–5 partners and 6 or more partners in the past six months were both more likely to report current PrEP use than participants who reported having zero or one partner; this association was significant for those with 6+ partners (aPR 2.28; 95% CI: 1.10–4.75). In addition, participants who had ever had an STI were more likely to report current PrEP use compared with those who had not (aPR = 1.56; 95% CI: 1.05–2.32).

For condom use in general as well as condom use during sex work, the relationship between current PrEP use and frequency of condom use was more complicated. Those who only sometimes used condoms were more likely to be currently taking PrEP compared with those who always used condoms, while those who never used condoms trended less likely to report current PrEP use (condom use, sometimes: aPR = 1.58; 95% CI: 1.05–2.36; condom use, never: aPR: 0.91; 95% CI: 0.48–1.72). We observed a similar trend for condom use during sex work (Figure 1).

## Discussion

We identified a variety of correlates of current PrEP use among transgender women of color who reported taking GAHT. Access to gender-affirming care was associated with current PrEP use, as was high sexual risk, confirming that both healthcare accessibility and risk awareness are related to the adoption of appropriate HIV prevention strategies among TWOC. Our study on current PrEP use amongst those reporting GAHT use adds to the literature on PrEP uptake in TWOC with respect to intersecting identities, individual sociodemographic characteristics, interpersonal behavioral characteristics, and structural characteristics.

We observed variation in the relationship between current PrEP use and different facets of gender, ethnicity, and sociocultural context. For example, we found that participants who primarily spoke Spanish at home were significantly more likely to report current PrEP use compared with those who primarily spoke English at home. This may reflect the successful tailoring of HIV prevention services to Spanish-speaking populations in New York City. In contrast, we did not observe an association between current PrEP use and nativity or race (with the exception of those identifying as multiracial); other researchers have explored PrEP use amongst TWOC taking GAHT and found it to be partially attenuated by race/ ethnicity (Rivera et al., 2023).

Individuals residing in Single-Room Occupancy (SRO) housing were significantly more likely to report current PrEP use than those with other housing arrangements. SROs may serve as bridges to a range of resources, including healthcare. Several of the largest supportive housing facilities in New York City offer intensive case management services and/or on-site medical facilities which may connect individuals to HIV preventive care (Bauman et al., 2013). Further, some supportive housing facilities specifically cater to individuals living with HIV/AIDs and may therefore also effectively support at-risk populations in accessing HIV resources (Lee et al., 2018; Remien et al., 2015).

We also found that those identifying outside of the gender binary were less likely to report current PrEP use. While all participants in the sample identified as transgender women and were taking gender-affirming hormone therapy, participants identifying with an additional gender identity may have had unique difficulties accessing PrEP because of their gender presentation. Healthcare provider insensitivity and hostility are associated with being perceived as gender nonconforming or not “passing” as one’s identified gender (Xavier et al., 2013). As a result, interpersonal stigma toward gender nonconformity may discourage engagement with healthcare providers and limit access to PrEP. Another possible explanation is that the messaging disseminated with current HIV prevention interventions may not be affirming of, or designed for, individuals identifying outside of the gender binary.

Another predictor of current PrEP use in our study of TWOC reporting GAHT use was access to gender-affirming healthcare and/or a primary care provider knowledgeable about transgender health. This aligns with previous research that has found that access to gender-affirming healthcare is a strong predictor for PrEP use among transgender women (Hood et al., 2018; Rivera et al., 2023). Given that all participants in our study reported GAHT use, it is not surprising that most participants in our sample reported access to gender-affirming care. However, our findings suggest that the qualitative nature of care also matters: participants who reported ‘Great/Good’ access to gender-affirming care and/or that their PCP knows ‘Almost everything’ about transgender healthcare were more likely to report current PrEP use, suggesting that the quality, competence, and affirmation of care matter in addition to access.

Finally, some, but not all, sexual risk behaviors predicted current PrEP use. We found a dose-response relationship between reported number of sexual partners over the past six months and likelihood of current PrEP use; we also found an association between ever having a sexually-transmitted infection and PrEP use. These findings are consistent with other research that finds that people who are more susceptible to HIV are more likely to use PrEP (Morgan et al., 2018), as well as with qualitative findings that individuals who have more sexual partners are aware of their own risk of HIV and know that they would benefit from PrEP (Mimiaga et al., 2014). Our results suggest that the relationship between self-perceived HIV risk and PrEP use may be broader than awareness of the number of sexual partners alone. On the other hand, condom use (or lack thereof) was not associated with current PrEP use. Multiple dimensions of risk perception may therefore contribute to the decision to take PrEP among TWOC taking GAHT.

### Strengths and limitations

As one of the few large cohorts of transgender women of color, the TURNNT cohort allowed us to observe predictors of current PrEP use among those who take GAHT that are unique to this population. Within this population, we were also able to examine PrEP use amongst those who identified as an additional gender (e.g., femme non-binary), an oft-ignored subpopulation that we found was less likely to report current PrEP use. That said, because within the broader TURNNT cohort we focused on the subset of those not living with HIV and on GAHT, our sample size was small. While we found many predictors of current PrEP use among those reporting GAHT use, small cell counts made it difficult to detect associations. We observed a number of non-statistically significant trends that could be further investigated with a larger sample size powered to potentially detect additional predictors of PrEP use among those reporting GAHT use. Additionally, while we controlled for age, sexual orientation, income, and housing stability, there may be residual confounding biasing our results.

The effect that we estimated in our study may not be generalizable to all TWOC in New York City, given our convenience sampling methodology. Our use of convenience sampling may also explain why we observed a higher-than-expected prevalence of GAHT and PrEP use, as some participants were referred from community health clinics. In addition, because nearly all participants in the original TURNNT cohort were using GAHT, we were unable to meaningfully compare PrEP use among those reporting GAHT use with those without GAHT. While we are able to examine characteristics of PrEP use amongst those reporting GAHT use, this limited our ability to tell whether predictors of PrEP use were consistent with different configurations of GAHT and PrEP use. Purposeful sampling may be employed in future cohorts of TWOC to further explore these associations in follow-up studies.

## Conclusions

Our findings suggest that multilevel factors— including individual-level characteristics (e.g., language spoken at home, housing type, religiosity), interpersonal characteristics (e.g., condom use), and structural characteristics (e.g., provider knowledge and willingness to provide transgender care) substantially influence PrEP use amongst TWOC reporting GAHT use. These results highlight the need for multilevel interventions that address structural and interpersonal determinants of health access and equity. Further, self-perceived risk may drive uptake of risk reduction strategies in this population, which could guide future chronic disease prevention efforts. Future research should include non-binary people in studies of transgender health, and researchers should consider how HIV prevention strategies can be adapted to affirm not only binary transgender individuals, but a broad range of gender identities.

## Supplementary Material

Supp 1

Supplemental data for this article can be accessed online at https://doi.org/10.1080/26895269.2026.2663935.

## Figures and Tables

**Table 1.** Sociodemographic characteristics, the TURNNT cohort

Age	Total N	%

18 to 24	21	15.7
25 to 34	49	36.6
35 to 44	48	35.8
45 to 55	16	11.9
Unknown	2	1.5
Income		
No income or <$10,000	57	41.9
$10,000–$29,999	35	25.7
> $30,000	23	16.9
Unknown	21	15.4
Race/ethnicity		
Asian/Asian American	3	2.2
Black/African American	34	25.0
Hispanic/Latina	63	46.3
Middle Eastern/Native American	1	0.7
Multiracial/Biracial	33	24.3
American Indian	1	0.7
Something Else	1	0.7
Nativity (US born)		
No	54	39.7
Yes	81	59.6
Unknown	1	0.7
Sexual orientation		
Straight	95	69.9
Non-straight	37	27.2
Don’t Know	4	2.9
Education		
High school graduate/GED	46	33.8
Less than high school	33	24.3
More than high school	57	41.9
Food insecurity	Total N	%
No	59	43.4
Yes	75	55.1
Unknown	2	1.5
Sex work		
No	33	24.3
Yes	102	75.0
Unknown	1	0.7
Current housing		
House	12	8.8
Apartment	82	60.3
Single room occupancy	27	19.9
Homeless shelter or drop-in	14	10.3
Primary care access		
No	27	19.9
Yes	109	80.1
Intimate partner violence		
No	59	43.4
Yes	70	51.5
Unknown	7	5.1
Sexual assault		
No	85	62.5
Yes	42	30.9
Unknown	9	6.6
