# Supplementary material for: Correlates of pre-exposure prophylaxis amongst transgender women of color taking gender-affirming hormone therapy: Findings from the TURNNT cohort study
Source: Int J Transgend Health. Author manuscript; Available in PMC 2026 Aug 8. (PMC13451024; doi:10.1080/26895269.2026.2663935)
Supplement: Supplementary file 1 [file NIHMS2178951-supplement-Supp_1.docx]

Supplementary Table 1. Sociodemographic characteristics, TURNNT cohort

| **Characteristic** | **N** | **%** |
| --- | --- | --- |
| Nativity (US Born) |  |  |
| No | 54 | 39.7 |
| Yes | 81 | 59.6 |
| Unknown | 1 | 0.7 |
| Language spoken at home |  |  |
| English | 67 | 49.3 |
| Spanish | 53 | 39.0 |
| English/Spanish Equally | 13 | 9.6 |
| Other | 3 | 2.2 |
| Religiosity |  |  |
| Very Religious | 21 | 15.4 |
| Somewhat religious | 31 | 22.8 |
| A little religious | 46 | 33.8 |
| Not religious at all | 35 | 25.7 |
| Unknown | 3 | 2.2 |
| Current Housing |  |  |
| House | 12 | 8.8 |
| Apartment | 82 | 60.3 |
| Room | 27 | 19.9 |
| Homeless shelter or drop-in | 14 | 10.3 |
| Other | 1 | 0.7 |
| Education |  |  |
| High school graduate/GED | 46 | 33.8 |
| Less than High School | 33 | 24.3 |
| More than High School | 57 | 41.9 |
| Age |  |  |
| 18 to 24 | 21 | 15.7 |
| 25 to 34 | 49 | 36.6 |
| 35 to 44 | 48 | 35.8 |
| 45 to 55 | 16 | 11.9 |
| Unknown | 2 | 1.5 |
| Race/Ethnicity |  |  |
| Asian | 3 | 2.2 |
| Black | 34 | 25.0 |
| Latinx | 63 | 46.3 |
| MENA | 1 | 0.7 |
| Multi | 33 | 24.3 |
| Native | 1 | 0.7 |
| Other | 1 | 0.7 |
| Income |  |  |
| No income or <$10,000 | 57 | 41.9 |
| $10,000 - $29,999 | 35 | 25.7 |
| > $30,000 | 23 | 16.9 |
| Unknown | 21 | 15.4 |
| Food Insecurity in Past 6 months |  |  |
| No | 59 | 43.4 |
| Yes | 75 | 55.1 |
| Unknown | 2 | 1.5 |
| History of sex work |  |  |
| No | 33 | 24.3 |
| Yes | 102 | 75.0 |
| Unknown | 1 | 0.7 |
| History of incarceration |  |  |
| No | 71 | 52.2 |
| Yes | 64 | 47.0 |
| Unknown | 1 | 0.7 |

Supplementary Table 2. Health characteristics, TURNNT cohort

| **Characteristic** | **N** | **%** |
| --- | --- | --- |
| Health Over Past 6 Months |  |  |
| Excellent or Very Good | 36 | 26.5 |
| Good | 48 | 35.3 |
| Fair or Poor | 52 | 38.2 |
| Have a Primary Care Physician |  |  |
| No or only some of past 6mo | 27 | 19.9 |
| Yes, all of past 6mo | 109 | 80.1 |
| Insurance |  |  |
| No or only some of past 6mo | 21 | 15.4 |
| Yes, all of past 6mo | 115 | 84.6 |
| Unmet Medical Need Over Past 6 Months |  |  |
| No | 100 | 73.5 |
| Yes | 35 | 25.7 |
| Unknown | 1 | 0.7 |
| PCP Knowledge of Trans Issues |  |  |
| Know almost everything | 53 | 42.1 |
| Know most things | 41 | 32.5 |
| Know some or little | 31 | 24.6 |
| Don't know | 1 | 0.8 |
| Insurance ever denied trans care |  |  |
| No | 77 | 62.1 |
| Yes | 40 | 32.3 |
| Don't Know or have never asked | 7 | 5.6 |
| Doctor ever denied trans care |  |  |
| No | 109 | 80.1 |
| Yes | 26 | 19.1 |
| Don't Know | 1 | 0.7 |
| Access to Trans Care |  |  |
| Great | 45 | 33.1 |
| Good | 52 | 38.2 |
| OK | 29 | 21.3 |
| Poor/Non-existent | 10 | 7.4 |
| Access to Trans Care (Dichotomous) |  |  |
| Great/Good | 97 | 71.3 |
| Ok/Poor | 39 | 28.7 |
| Have a Doctor for Trans Care |  |  |
| No | 10 | 7.4 |
| Yes | 125 | 91.9 |
| Unknown | 1 | 0.7 |

Supplementary Table 3. Gender/sexuality characteristics, TURNNT cohort

| **Characteristic** | **N** | **%** |
| --- | --- | --- |
| Gender Identity |  |  |
| Transgender woman | 107 | 78.7 |
| Other | 29 | 21.3 |
| Sexual Orientation |  |  |
| Straight | 95 | 69.9 |
| Non-straight | 37 | 27.2 |
| Don't Know | 4 | 2.9 |
| Living as Trans Full-time |  |  |
| Some or none of the time | 10 | 7.4 |
| All of the time | 126 | 92.6 |
| Legal Name Change |  |  |
| No | 39 | 28.7 |
| Yes | 97 | 71.3 |
| Legal Gender Marker Changed |  |  |
| No | 42 | 30.9 |
| Yes | 94 | 69.1 |
| Age at Trans Identity |  |  |
| < 18 | 46 | 34.1 |
| 18-24 | 56 | 41.5 |
| >24 | 33 | 24.4 |
| Unknown | 1 | 0.7 |
| Breast Augmentation |  |  |
| No | 63 | 46.3 |
| Yes | 65 | 47.8 |
| Unknown | 8 | 5.9 |
| Silicone Injections/Implants |  |  |
| No | 102 | 75.0 |
| Yes | 28 | 20.6 |
| Unknown | 6 | 4.4 |
| Bottom Surgery |  |  |
| No | 98 | 72.1 |
| Yes | 18 | 13.2 |
| Unknown | 20 | 14.7 |
| Facial Feminization Surgery |  |  |
| No | 95 | 69.9 |
| Yes | 35 | 25.7 |
| Unknown | 6 | 4.4 |

Supplementary Table 4. Behavioral characteristics, TURNNT cohort

| **Characteristic** | **N** | **%** |
| --- | --- | --- |
| Frequency of Alcohol Use |  |  |
| Several/week to daily | 24 | 17.6 |
| Few times/month | 36 | 26.5 |
| Once every few months | 25 | 18.4 |
| Once or twice total | 17 | 12.5 |
| Never | 33 | 24.3 |
| Unknown | 1 | 0.7 |
| Drug Use |  |  |
| No | 86 | 63.2 |
| Yes | 50 | 36.8 |
| Frequency of Drug Use |  |  |
| Weekly or more | 11 | 33.3 |
| A few times a month or less | 22 | 66.7 |
| Ever Experienced Intimate Partner Violence |  |  |
| No | 59 | 43.4 |
| Yes | 70 | 51.5 |
| Unknown | 7 | 5.1 |
| Ever Experienced Sexual Assault |  |  |
| No | 85 | 62.5 |
| Yes | 42 | 30.9 |
| Unknown | 9 | 6.6 |
| Ever Had Gonorrhea, Syphilis, or Chlamydia |  |  |
| No, never | 73 | 53.7 |
| Yes | 63 | 46.3 |
| # of Sexual Partners in Past 6mo |  |  |
| None | 19 | 14.0 |
| One person | 38 | 27.9 |
| 2-5 people | 40 | 29.4 |
| 6+ people | 36 | 26.5 |
| Don't Know | 3 | 2.2 |
| Condom Use |  |  |
| Always | 49 | 41.2 |
| Some of the time | 41 | 34.5 |
| Never | 29 | 24.4 |
| Sex Work Frequency |  |  |
| Weekly or more | 36 | 65.5 |
| A few times a month or less | 19 | 34.5 |
| Condom Use During Sex Work |  |  |
| Always | 31 | 56.4 |
| Sometimes or never | 24 | 43.6 |
| Sexual Risk (Past 6mo) |  |  |
| Lowest: <6 Partners, Condom Use | 48 | 36.1 |
| Moderate: <6 Partners, Some/No Condom Use | 49 | 36.8 |
| Moderate: 6+ Partners, Condom Use | 16 | 12.0 |
| Highest: 6+ partners, Some/No Condom Use | 20 | 15.0 |
